# Effect of aerobic exercise on the improvement of executive function in children with attention deficit hyperactivity disorder: a systematic review and meta-analysis

**DOI:** 10.3389/fpsyg.2024.1376354

**Published:** 2024-06-04

**Authors:** Gao Yang, Qiang Liu, Wei Wang, Wei Liu, Junfeng Li

**Affiliations:** ^1^College of Sports and Health, Medicine & Technology College of Zunyi Medical University, Zunyi, China; ^2^Institute of Motor Quotient, Southwest University, Chongqing, China; ^3^Department of Physical Education, Central South University, Changsha, China; ^4^School of Physical Education and Health, Zunyi Medical University, Zunyi, China; ^5^Institute of Physical Education, Xuzhou Kinder Garten Teachers College, Xuzhou, China; ^6^Ministry of Sports, Shandong Technology and Business University, Yantai, China

**Keywords:** aerobic exercise, ADHD, executive functions, children, meta-analysis

## Abstract

**Objective:**

Aerobic exercise (AE) interventions are beginning to be used as an emerging adjunctive treatment modality in the treatment of children with Attention Deficit Hyperactivity Disorder (ADHD). However, to date, there is no substantial evidence to support the improved effects of aerobic exercise intervention in children with ADHD aged 6–12 years. This study aims to investigate the effect of aerobic exercise therapy on executive function in children with attention deficit hyperactivity disorder aged 6–12 years.

**Method:**

We conducted a systematic review and meta-analysis using PubMed and Web of Science. The cut-off date was June 1, 2023. The aim was to assess the impact of aerobic exercise interventions on children with ADHD and all randomized controlled trials eligible for aerobic exercise interventions for children with ADHD were included. Nine randomized controlled trials were screened for eligibility for systematic evaluation, and the nine studies were assessed for risk of bias using the PEDro score and the GRADE Quality of Evidence Evaluation System for quality grading of outcome indicators. After testing for heterogeneity, a random-effects model was selected for analysis. Finally, meta-analyses and regression analyses were performed on the core functions (inhibitory control, cognitive flexibility, and working memory) and subgroups of the nine studies on executive function using Revman 5.4 and Stata 16.0.

**Results:**

The risk of bias evaluation showed a mean PEDro score of 7.78, and of the nine studies, two were rated as having excellent methodological quality, while the remaining seven had a good level of evidence, and the GRADE evidence evaluation showed that the outcome indicators were all of moderate quality. Inhibitory control [SMD = 0.83,95% CI (0.37–1.29), Z = 3.51, *p* = 0.0005], cognitive flexibility [SMD = 0.65,95% CI (0.37–0.93), Z = 4.58, *p* < 0.00001], and working memory [SMD = 0.48,95% CI (0.02–0.95), Z = 2.03, *p* = 0.04] were statistically significant, with effect sizes of moderate or higher; furthermore, in subgroup analyses type of intervention, duration, intensity, and medication use had different effects on inhibitory control and cognitive flexibility, and the combined IC, CF statistic found that a single category of aerobic exercise ( *β* = 0.867, *p* < 0.001), moderate intensity ( *β* = 0.928, *p* < 0.001), 6–12 weeks (β = 0.804, *p* < 0.001), 60-90 min ( *β* = 0.894, *p* < 0.001), and the use of medication ( *β* = 1.202, *p* = 0.002) were better for overall improvement in EF.

**Conclusion:**

Aerobic exercise therapy significantly improved executive functioning in children with ADHD, showing above moderate effect sizes especially in inhibitory control, cognitive flexibility, and working memory. Aerobic exercise therapy can be used as a reference in improving executive function in children with ADHD, but given the limitations of this study, it should be used with caution when applied in clinical settings.

## 1 Introduction

Attention deficit hyperactivity disorder (ADHD) is one of the most common neurodevelopmental disorde (Brassell et al., 2017; Thapar et al., 2017; Du Rietz et al., 2021). The prevalence of ADHD is approximately 5.29% worldwide (Posner et al., 2020). Up to 7.2% of children have ADHD and the symptoms of ADHD affect 60% of children into adulthood (Thomas et al., 2015; Sibley et al., 2017). According to the Diagnostic and Statistical Manual of Mental Disorders, fifth edition, people with ADHD mainly present with symptoms of hyperactivity, impulsivity, and inattention (American Psychiatric Association, 2013), often with a learning disorder, conduct disorder, or coexisting oppositional defiant disorder mental illnesses (Biederman and Faraone, 2005). Inconsistency with normal developmental levels can cause various problems in academic and social activities in children with ADHD (American Psychiatric Association and DSM Task Force, 2017), resulting in learning difficulties, impaired social skills, and strained family–parent relationships (Harpin, 2005), such as difficulty completing studies, lower social skills, and increased family conflict. In addition, they are more likely to develop comorbidities such as depression, anxiety, and developmental coordination disorders (Barnard-Brak et al., 2011).

Executive functioning (EF) is the regulation of basal cognition through top-down higher mental processes that are essential for reaching goal-directed adaptive behaviors and maintaining attention (Diamond, 2013, 2020). The core symptoms of ADHD stem from major deficits in EF, with most patients exhibiting one or more such deficits (Nigg et al., 2005; Willcutt et al., 2005). The key components of EF, including control over updating the content of working memory (WM), shifting (i.e., task switching), and inhibitory control (IC), form the infrastructure of EF (Miyake et al., 2000). Central to the psychological and behavioral characteristics of ADHD patients and closely related to their performance in school, work, and social settings are the three core functions of IC, cognitive flexibility (CF), and WM. Among them, cognitive flexibility is mainly divided into two subtypes: reactive and spontaneous (Eslinger and Grattan, 1993), reactive CF refers to an individual’s ability to quickly adjust his or her thinking strategies and behavioral patterns in response to changes in the external environment, whereas spontaneous CF refers to an individual’s ability to generate a diverse range of new ideas on his or her own without external pressures to make changes, explore the unknown and the ability to adjust their thinking (Ebersbach and Hagedorn, 2011). Deficits in these three core functions are directly linked to the everyday performance of ADHD patients, such as the extent of their learning difficulties, social challenges, and behavioral problems (Diamond, 2013), which play an important role in ADHD patients’ physical and mental health, behavioral management, learning efficacy, and social interaction (Diamond, 2013; Mueller et al., 2017). Thus, improvement of EF in children with ADHD is extremely important. Commonly used treatments use medication (methylphenidate or atomoxetine) or behavioral interventions (Tantillo et al., 2002; Barnard-Brak et al., 2011), and traditional treatments are expensive, making them unaffordable for patients’ families (Cornelius et al., 2017). It also has potential risks such as slowed growth, appetite suppression, abdominal pain, headache, and difficulty sleeping (Swanson et al., 2007; Newcorn et al., 2010), and lacks significant long-term effects (Molina et al., 2009; Cornelius et al., 2017).

An alternative intervention is aerobic exercise (AE). AE is a physical activity in which inhaled oxygen adequately meets the body’s energy requirements during exercise through aerobic metabolism (William, 2006) by performing low-to-high-intensity movements for long periods (Sharon, 2007). In recent years, numerous studies have found (Liu et al., 2022) that improvement in EF deficits in children with ADHD can be achieved with AE (Chang et al., 2014; Brassell et al., 2017; Miklos et al., 2020). Chang et al. (2014), Brassell et al. (2017), and Miklos et al. (2020), conducted trials with aerobic fitness (Brassell et al., 2017), swimming exercise (Chang et al., 2014), and running exercise (Miklos et al., 2020) respectively, and found significant improvement in IC or CF. In addition, some studies have found that cerebral oxygenation and cerebral blood volume improve at higher levels of exercise intensity, which in turn lead to improvement in prefrontal-dependent cognitive performance (Gondoh et al., 2009; Hwang et al., 2016). However, in Diamond and Ling (2019), AE and resistance intervention training were considered the least effective among the methods to improve executive function (Diamond and Ling, 2019), and similarly, another study concluded that AE had no significant effect on EF improvement (Takacs and Kassai, 2019).

Currently, most research has focused on exploring the effects of exercise interventions on ADHD improvement (Cerrillo-Urbina et al., 2015; Seiffer et al., 2022), and only some have explored the improvement effect of exercise interventions on EF (Welsch et al., 2020; Sung et al., 2022). Moreover, some found positive evidence of improvement after reviewing AE interventions in children or adolescents with ADHD in subgroups (Liang et al., 2021). However, both randomized controlled trials (RCT) and non-RCT experimental studies were included in these studies, which also included children, adolescents, and adults of different ages. Only a few explored the effects of AE on IC, CF, and WM core functions in EF (Liang et al., 2021). Thus, there is a lack of a systematic evaluation of the effectiveness of AE interventions to improve IC, CF, and WM functioning in 6–12-year-old children with ADHD. Therefore, the main objectives of this review were (1) to systematically evaluate the effects of AE on core functions in EF in ADHD patients aged 6–12 years and (2) to explore the effects of AE intervention type, intensity, medications taken, duration and periodicity on the improvement of EF in children with ADHD.

Therefore, the participants in this study comprised children aged 6–12 years diagnosed with ADHD. Children in this age group are the key target of ADHD intervention research and effective interventions are particularly important for their learning and social development. The interventions were AE in either a combination of movements or a single event, including AE with added neurocognitive training. To accurately assess the effects of AE on EF in children with ADHD, we compared an experimental group undertaking an AE intervention with a control group undertaking conventional or other exercise treatments. We then measured the effects of AE on EF in children with ADHD, particularly IC, CF, and WM, and their significance in subgroup analyses. The study design was based on an RCT of an ADHD intervention to ensure the validity and reliability of the findings and minimize bias through random assignment.

Our systematic review and meta-analysis aimed to evaluate the effects of AE on EF in children with ADHD aged 6–12 years and provide more definitive intervention guidelines for this group of children. Analyzing the effect of AE on the improvement of core functions in EF should not only enhance the findings in the literature, but also provide a scientific basis for the adjunctive treatment of EF in children with ADHD. In addition, this study explores the effects of the type, intensity, medication taken, and time and period of the AE intervention on the improvement effect of EF, further refining the application of AE interventions used in ADHD treatment.

## 2 Methods

In this study, we did not pre-register for meta-analysis. We will do our best to ensure the credibility and transparency of the study by providing a detailed description of the study methodology, interpretation of results, and data sharing. Readers are welcome to share any questions or suggestions regarding our research methods and are willing to provide further explanations and discussions.

### 2.1 Search strategy

In this study, a systematic evaluation and meta-analysis were conducted according to the criteria specified in the Preferred Reporting Items for Systematic Reviews and Meta-Analyses (PRISMA) guidelines (Liberati et al., 2009). Literature searches were conducted in the PubMed and Web of Science databases with a publication deadline of June 1, 2023. To search for relevant topics, titles, and abstracts, the following keywords were used in both databases: “children,” “physical activity,” “exercise,” “running,” “cycling,” “swimming,” “executive functioning,” “ADHD,” and “attention deficit hyperactivity disorder.” For example, the following search strategy was used in Web of Science: #1: (TS = (“ADHD” OR “Attention Deficit Disorder with Hyperactivity”)); #2: (TS = (“aerobic exercise” OR “physical activity” OR “exercise” OR “children” OR “running” OR “cycling” OR “swimming”)); #3: (TS = (“executive function”)); and #4: #1–#3. Additionally, the literature was searched and screened by two reviewers (GY and HS) independently.

### 2.2 Selection criteria and screening

Studies eligible for inclusion in this study were selected using the following criteria: (1) study design: RCT based on ADHD intervention; (2) study population: children aged 6–12 years with confirmed ADHD; (3) type of intervention: the experimental group used a combination of movement-based or single-item AE (e.g., “Shape Up,” combined aerobic and neurocognitive, cycling, running, table tennis, swimming AE, etc.) as interventions in the study, and the control group was treated with conventional or other exercise treatment; (4) Intervention time: the experimental group conducted more than one aerobic exercise session; (5) Outcome indicators: using tools related to task testing (e.g., Tower of London, Digit Span Forward, Backward Test, Go-No-Go Task, Flanker Task Stroop Task, Trail Making Task, Wisconsin Card Sorting Test) to investigate inhibition switching, CF, and WM indicators in EF in children with ADHD.

Exclusion criteria for this study: (1) literature not written in English; (2) case reports; (3) reviews; (4) conference abstracts; (5) book chapters or other; (6) studies containing other case patients; (7) exclusion of peer-reviewed articles.

### 2.3 Data extraction

Two reviewers (GY and QL) extracted EF test data from the full text of the included studies, and issues of disagreement were discussed and resolved by two other reviewers (QL and JL). Basic characteristics of children with ADHD (mean age in different groups, gender/number), experimental site, exercise intensity, medication use or not, intervention design (type of AE, intervention period, intervention frequency, intervention intensity), and outcome indicators (IC, CF, WM) of the task tests were extracted from the included literature. As well as extracting data on the occurrence of adverse events, this includes, but is not limited to, any physical discomfort, injuries, or other health problems that may result from participation in aerobic exercise. We extracted the mean (Mean) and standard deviation (SD) of each task test performed before and after the intervention in the experimental and control groups of ADHD children with EF from the included studies, while recording the sample size (*n*) of the two groups and performing meta-analysis of raw scores of the different task tests (e.g., color-word, the sum of correct responses, non-perseverative errors, total correct, accuracy, etc.), for meta-analysis. If the exact data were not provided in the article, they were obtained by contacting the authors, or they could be calculated from the data provided in the article. In addition, if experimental data were reported in graphical form in the article, Get Data Graph Digitizer software was used to measure the data to obtain the mean and SD of the test before and after the two groups.

### 2.4 Data analysis

The amount of change in inhibitory control, cognitive flexibility and working memory from pre to post intervention was extracted, and if the original study directly reported means and standard deviations for the outcome variables, these reported values could be used directly. If the original studies did not directly report means and standard deviations for the outcome variables, we then needed to take Corr as 0.40 or 0.50 according to the guidelines in section 16.1.3.2 of the Cochrane Handbook 5.0.2 and follow the Mean E, change = Mean E, final-Mean E, baseline, $SD\;E,\;\mathrm{change}=\sqrt{{SD}_{E,\mathrm{baseline}}^{2}+{SD}_{E,\mathrm{final}}^{2}-\left(2*\mathrm{Corr}*{SD}_{E,\mathrm{baseline}}*{SD}_{E,\mathrm{final}}\right)}$ formula to calculate the mean and standard deviation of the pre- and post-intervention outcome variables to meet the data requirements needed to perform Meta-analysis. Meta-analysis as well as meta-regression analyses were then performed using Review Manager Software V.5.4 and stata/map 16.0 on the means and standard deviations extracted for changes in outcome variables before and after the intervention. Standardized mean difference (SMD) was used as the combined statistic for this study due to the different outcome indicators of the test instruments, and 95% confidence interval was used as the combined data effect (Liu et al., 2022). For data on adverse events, descriptive statistics will be used to summarize the reporting of adverse events in the included studies. To improve the robustness of the results, we analyzed the data using a random-effects model, which more accurately reflects differences in effects across studies and provides a more representative estimate of the combined effect. And Hedges’ g method was used to quantify the effect sizes of the exercise interventions, which were categorized into three classes according to the common criteria for effect size estimation: a small effect of 0.2 at least less than 0.5, a medium effect of 0.5 at least less than 0.8, and a large effect of 0.8 and above (Hanley et al., 2003). After combining the statistics, if *p* < 0.05, there was statistical significance; if *p* > 0.05, there was no statistical significance.

### 2.5 Qualitative analysis

In this study, to ensure the reliability of the cited evidence, the PEDro scoring system was used to evaluate the risk of bias of the nine selected papers (Yang et al., 2022). The PEDro scoring system consists of eleven criteria: explicitly setting the inclusion criteria for the study participants, the randomization of the participant selection process and the concealment of the allocation in the grouping, the similarity at baseline and blinding of participants, therapists, and evaluators, a follow-up rate of greater than 85%, the use of intention-to-treat analysis, statistical comparisons between groups, and accurate reporting of data points and indicators of variability are all key factors in ensuring the reliability and validity of the study results. According to the PEDro scale, each item is scored out of 1 and is based on a yes or no response, with scores between 6–8 considered good, 9–10 considered excellent, 4–5 considered acceptable, and less than 4 indicating quality of evidence. 4 indicated that the evidence was of low quality (Maher et al., 2003). Two independent reviewers (GY and QL) were responsible for performing the assessment of the risk of bias of the literature included in the study, and any disagreements were resolved by consulting the corresponding author (JL).

### 2.6 Assessment of the certainty of evidence

GRADE (Grading Recommendations to Assessment Development and Evaluation system) is a system for categorizing and evaluating the quality and strength of evidence, which is one of the international standards today (Liu et al., 2020). The studies included in the study were RCTs with high-grade evidence, and we rated the level of evidence based on five aspects: risk of bias, indirectness, inconsistency, imprecision, and publication bias (Zhang et al., 2019). The quality of evidence was graded in 4 levels of high (high), moderate (moderate), low (low), and very low (very low) for the main outcome indicators (IC, CF, and WM).

## 3 Results

### 3.1 Identification of studies

**Figure 1:**
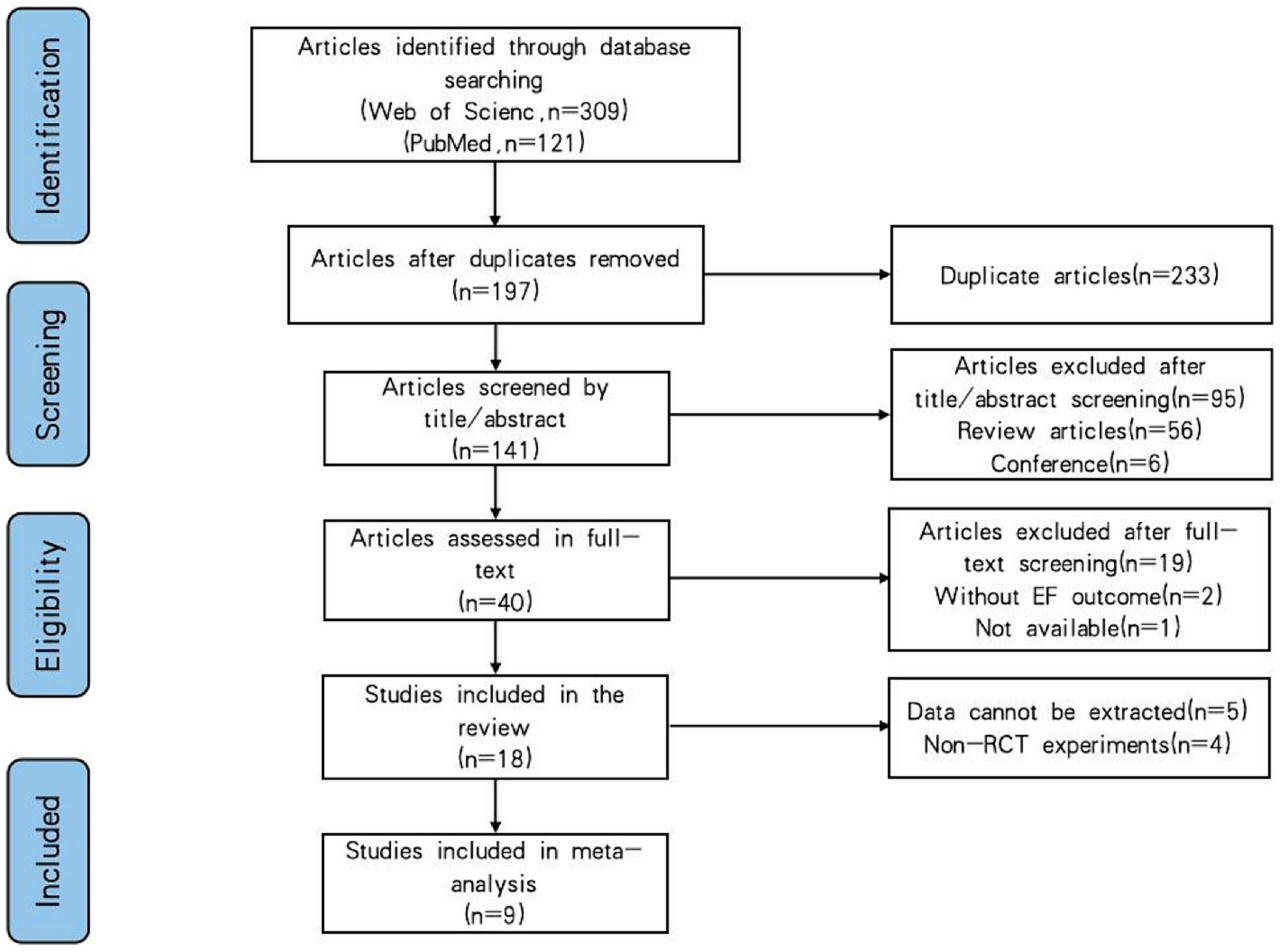
Flowchart of the selected literature.

We retrieved 309 studies from Web of Science data and 121 studies from the PubMed database. Two reviewers (GY and QL) went through a rigorous screening process, excluding studies unrelated to the study design after the title and abstract screening, and removing duplicate studies, review articles and conference-type studies. Forty potential studies were entered into the full-text evaluation screening, 19 unrelated studies were excluded after the full-text screening, and two studies without EF outcome indicators were excluded. Eighteen studies were screened for full-text review, data from 5 studies were excluded without contacting the authors, and four studies that were AE interventions but not RCT trials were excluded. After the screening, nine studies met the criteria for inclusion in the systematic evaluation and meta-analysis, and the detailed study screening process is shown in Figure 1.

### 3.2 Description of included studies

A total of nine RCT trials were included in this study for analysis, and Table 1 shows the relevant characteristics of the included studies. The studies involved 377 children with ADHD (300 boys and 77 girls), with a mean age ranging from 9.31 ± 1.33 years; 133 of the children with ADHD used medication, with one study not reporting medication use (Nejati and Derakhshan, 2021). The trials included in this study were from 3 different countries, and all studies were conducted in Asia and Europe, namely China (Chang et al., 2012, 2022; Pan et al., 2015, 2016; Liang et al., 2022), Switzerland (Benzing et al., 2018; Benzing and Schmidt, 2019), and Iran (Memarmoghaddam et al., 2016; Nejati and Derakhshan, 2021).

**Table 1:** Characteristics of included trials in this review.

Author, year (country)	N (M/F)	Age (M ± SD)	RCT design	Drug use	Measurement stools/indicator	Intensity	Intervention method	Exercise categories	Frequency of exercise	Main effect
Pan et al. (2016) (Taiwan, China)	*N* = 32 (M = 32/F = 0)	EG:8.93 ± 1.49 CG:8.87 ± 1.56	NR	EG = 7 CG = 7	IC:ST	MI	EG: table tennis exercise CG: Not receiving new sport activities	SAE	12 weeks, 2 times/week,70 min/each	IC:RS improves ( *p* < 0.01)
Benzing et al. (2018) (Switzerland)	*N* = 46 (M = 38/F = 8)	EG:10.46 ± 1.35 CG:10.50 ± 1.41	SB	EG = 19 CG = 17	IC:FT CF:FT WM:CSBT	MVI	EG: “Shape Up “exergaming exercises CG: Watched a documentary report about mountain running	CAE	One session 15 min	IC:RT with potential to improve ( *p* < 0.05); CF:RT with potential to improve ( *p* < 0.05); WM:RS no improves ( *p* > 0.05)
Benzing and Schmidt (2019) (Switzerland)	*N* = 51 (M = 43/F = 8)	EG:10.46 ± 1.30 CG:10.39 ± 1.44	NB	EG = 20 CG = 17	IC:ST CF:FT WM: CSBT	MVI	EG: “Shape Up” exergaming exercises CG: Not receiving any treatment (waiting-list)	CAE	8 weeks, 3 times/week, 30 min/each	IC:RT improves ( *p* < 0.05); CF:RT improves ( *p* < 0.05); WM:RS no improvement ( *p* > 0.05)
Memarmoghaddam et al. (2016) (Iran)	*N* = 36 (M = 36/F = 0)	EG:8.31 ± 1.29 CG:8.29 ± 1.31	NB	EG = 0 CG = 0	IC:ST, GNGT	MVI	EG: Exercise program (such as football, basketball, for aerobic exercise) CG: Did not receive any intervention	CAE	8 weeks, 3 times/ week, 90 min/each	IC:RS and RT improves ( *p* < 0.05)
Pan et al. (2015) (Taiwan, China)	*N* = 30 (M = 30/F = 0)	EG:9.08 ± 1.43 CG:8.90 ± 1.66	NB	EG = 9 CG = 9	IC:ST CF:WCST	MI	EG: table tennis exercise CG: Not receive any intervention	SAE	12 weeks, 2 times/week, 70 min/each	IC:RS significantly improves ( *p* < 0.01) CF:RS improves ( *p* < 0.05)
Chang et al. (2022) (Taiwan, China)	*N* = 32 (M = 26/F = 6)	EG:8.31 ± 1.30 CG:8.38 ± 1.31	NR	EG = 4 CG = 4	IC:ST CF:WCST	MI	EG: Actual table tennis training CG: No additional training	SAE	12 weeks, 3 times/week, 60 min/each	IC:RS improves; CF:RS improves ( *p* < 0.01)
Nejati and Derakhshan (2021) (Iran)	*N* = 30 (M = 30/F = 0)	EG:9.43 ± 1.43 CG:9.43 ± 1.43	NR	EG = NR CG = NR	IC: GNGT CF: WCST WM: NBT	NR	EG: Exercise for cognitive improvement and rehabilitation (EXCIR) CG: Running	CAE	4–5 weeks, 3 times/week, 40–50 min/each	WM: Improved RS and response time ( *p* < 0.05); CF: RS improve ( *p* < 0.05); IC: RS improve ( *p* < 0.05), RT no difference ( *p* > 0.05)
Liang et al. (2022) (China)	*N* = 80 (M = 28/F = 52)	EG:8.37 ± 1.42 CG:8.29 ± 1.27	NR	EG = 0 CG = 0	IC: FT CF: TMT WM: TOLT	MVI	EG: Combined aerobic and neurocognitive-exercise CG: Without taking any additional exercise program or medical treatment	CAE	12 weeks, 3 times/week, 60 min/each	IC: RT improves, RS no significant effect ( *p* < 0.01); WM:RS no significant effect, RT improves ( *p* < 0.001); CF: RS no significant effect, RT improves ( *p* < 0.05)
Chang et al. (2012) (Taiwan, China)	*N* = 40 (M = 37/F = 3)	EG:10.42 ± 0.95 CG:10.4 ± 0.875	NB	EG = 10 CG = 10	IC:ST CF: WCST	MI	EG: run on a treadmill CG: Watched a running/exercise-related video	SAE	One session 30 min	IC:RS improves ( *p* < 0.05) CF:RS improves ( *p* < 0.05)

*N*, Number; M, Male; F, Female; M, Mean; SD, Standard Deviation; NR, No report; SB, Single-blind; NB, Non-blind; EG, experimental group; CG, control group; IC, inhibitory control; CF, cognitive flexibility; WM, working memory; ST, Stroop task; WCST, Wisconsin card sorting test; FT, flanker task; CSBT, color span backwards task; ST, Simon task; GNGT, go-no-go task; NBT, N-back test; TOLT, tower of london test; TMT, trail making test; MI, Moderate intensity; MVI, moderate to vigorous intensities; SAE, single aerobic exercise; CAE, combination aerobic exercise; RT, response time; RS, raw score.

Of the blinding of patients and trialists implemented in the nine included RCT studies, one was single-blinded (Benzing et al., 2018), four studies were unblinded (Chang et al., 2012; Pan et al., 2015; Memarmoghaddam et al., 2016; Benzing and Schmidt, 2019), and the remaining four study designs were not reported (Pan et al., 2016; Nejati and Derakhshan, 2021; Chang et al., 2022; Liang et al., 2022). Regarding intervention duration, two studies used a single 15–30 min intervention (Chang et al., 2012; Benzing et al., 2018) and the other seven studies were 4–12 weeks (Pan et al., 2015; Memarmoghaddam et al., 2016; Pan et al., 2016; Benzing and Schmidt, 2019; Nejati and Derakhshan, 2021; Chang et al., 2022; Liang et al., 2022). In terms of intervention frequency, studies were 2–3 times per week for 30–90 min each time. In addition, all nine studies performed the intervention as a single category of AE, combined with a combination of AE classes. Four studies used a single-category AE intervention (Chang et al., 2012, 2022; Pan et al., 2015, 2016), using table tennis (Pan et al., 2015, 2016; Chang et al., 2022), and running (Chang et al., 2012), respectively, and five studies used a combination category AE intervention (Memarmoghaddam et al., 2016; Benzing et al., 2018; Benzing and Schmidt, 2019; Nejati and Derakhshan, 2021; Liang et al., 2022), using a game console for “Shape Up” AE with a combination of 6 types of movements (Benzing et al., 2018; Benzing and Schmidt, 2019), AE with a combination of different sports (e.g., soccer, basketball, etc.) (Memarmoghaddam et al., 2016), and AE with a combination of movements designed to enhance cognitive performance (Nejati and Derakhshan, 2021; Liang et al., 2022). Among the nine RCT trials in the control group, there were five not receiving any treatment (Pan et al., 2015; Memarmoghaddam et al., 2016; Benzing and Schmidt, 2019; Chang et al., 2022; Liang et al., 2022), two studies conducted with Watch the sports video (Benzing et al., 2018; Chang et al., 2012) and 2 studies were conducted with running and not receiving new sport activities (Pan et al., 2016; Nejati and Derakhshan, 2021), and it is worth mentioning that in the study by Pan et al. (2016) both experimental and control groups performed aerobic exercise workouts, but there was no cognitive load in the control group’s exercise program. Participants’ heart rates were controlled within 50 to 80% of heart rate reserve (HRR) when they received the intervention in nine trials. Of these, intervention intensity was designated as moderate (moderate intensity) in 4 studies (Chang et al., 2012, 2022; Pan et al., 2015, 2016) and between moderate to vigorous intensities (moderate to vigorous intensities) in another 4 studies (Memarmoghaddam et al., 2016; Benzing et al., 2018; Benzing and Schmidt, 2019; Liang et al., 2022). In addition, one study did not report a specific intervention intensity in the original literature (Nejati and Derakhshan, 2021).

As shown in Table 2. The included RCT studies all used at least one or more EF testing instruments, with pre-and post-tests of IC, CF, raw scores of WM (correctness and error rate scores), and reaction time in the EF of children with ADHD. Of these, six studies used the Stroop Task, three studies used the Flanker Task and two studies used the Go-No-Go Task to test IC; two studies and four studies tested CF using the Flanker Task, Wisconsin Card Sorting Test, respectively; two studies used the Color Span Backwards Task, and two other studies used the N-Back Test, Tower of London Test to test WM.

**Table 2:** EF test characterization chart.

EF tests used	Main test indicators	Study	Adverse event
Stroop task	Inhibitory control	Pan et al. (2016), Benzing and Schmidt (2019), Memarmoghaddam et al. (2016), Pan et al. (2015), Chang et al. (2022), and Chang et al. (2012)	No
Flanker task	Inhibitory control cognitive flexibility	Benzing et al. (2018), Benzing and Schmidt (2019) and Liang et al. (2022)	No
Go-no-go task	Inhibitory control	Memarmoghaddam et al. (2016) and Nejati and Derakhshan (2021)	No
Wisconsin card sorting test	Cognitive flexibility	Pan et al. (2015), Chang et al. (2022), Nejati and Derakhshan (2021), and Chang et al. (2012)	No
Tower of London Test	Working memory	Liang et al. (2022)	No
N-Back test	Working memory	Nejati and Derakhshan (2021)	No
Color span backwards task	Working memory	Benzing et al. (2018), Benzing and Schmidt (2019)	No

In addition, we observed that no adverse events were reported in the experimental groups participating in aerobic exercise in all nine included studies. This observation emphasizes the reliability of aerobic exercise for children with ADHD in terms of safety. It can provide a solid theoretical basis for future research and evidence for clinical practice regarding the safety and efficacy of aerobic exercise interventions to complement other treatment options in improving executive function in children with ADHD.

### 3.3 Quality assessment

Table 3 presents the PEDro scoring details for the selected literature. For the nine randomized controlled trials (RCTs) involving aerobic exercise interventions, the average PEDro score was 7.78, indicating a high level of credibility. All nine studies were conducted as randomized controlled trials and were assessed for bias risk using the PEDro scoring system, resulting in respective PEDro scores. Among these nine studies, two were rated as having excellent methodological quality, while the remaining seven were assessed to have good evidence levels. It is noted that blinding of therapists and assessors was not reported in any of the nine studies, and only three studies reported blinding of participants (Pan et al., 2016; Benzing et al., 2018; Benzing and Schmidt, 2019). Overall, the literature included in this study demonstrated generally good methodological quality (Yao et al., 2021; Yong et al., 2021).

**Table 3:** Analysis of the methodological quality of the studies (PEDro scores).

Author (reference)	Item 1	Item 2	Item 3	Item 4	Item 5	Item 6	Item 7	Item 8	Item 9	Item 10	Item 11	Score
Pan et al., 2016	Y	Y	N	Y	Y	N	N	Y	Y	Y	Y	8
Benzing et al. (2018)	Y	Y	Y	Y	Y	N	N	Y	Y	Y	Y	9
Benzing and Schmidt (2019)	Y	Y	Y	Y	Y	N	N	Y	Y	Y	Y	9
Memarmoghaddam et al. (2016)	Y	Y	N	Y	N	N	N	Y	Y	Y	Y	7
Pan et al. (2015)	Y	Y	N	Y	N	N	N	Y	Y	Y	Y	7
Chang et al. (2022)	Y	Y	N	Y	N	N	N	Y	Y	Y	Y	7
Nejati and Derakhshan (2021)	Y	Y	N	Y	N	N	N	Y	Y	Y	Y	7
Liang et al. (2022)	Y	Y	Y	Y	N	N	N	Y	Y	Y	Y	8
Chang et al. (2012)	Y	Y	Y	Y	N	N	N	Y	Y	Y	Y	8

Range:0–11. Item 1: setting of appropriateness criteria; item 2: random assignment of participants; item 3: assignment concealment; item 4: similarity of groups at baseline; item 5: blinding of participants; item 6: blinding of therapists; item 7: blinding of evaluators; item 8: follow-up rate over 85%; item 9: use of intention-to-treat analyses; item 10: statistical comparisons between groups; and item 11: reporting of data points and indicators of variability.

### 3.4 Characteristics of executive functions task testing

The Stroop Test in this study primarily assessed IC by asking participants to name colors while ignoring the meanings of color words. In particular, in the Stroop Color-Word condition, participants were required to demonstrate strong inhibition when confronted with conflicting color words and colors (Pan et al., 2015).

The Simon Task assessed IC by asking participants to respond quickly to the color of the star on the screen. This task emphasized the ability to inhibit symmetrical side responses; in other words, participants needed to provide intuitive responses when the response buttons were not aligned with the stimulus location (Benzing and Schmidt, 2019).

The Go-No-Go Task was used to assess IC by asking participants to respond when confronted with certain stimuli (Go stimuli) and inhibit responses when confronted with other stimuli (No-Go stimuli) (Nejati and Derakhshan, 2021).

The Wisconsin Card Sorting Test (WCST) focuses on assessing transferential cognitive flexibility, which is the individual’s ability to adjust thinking strategies and behaviors in response to changes in goals or rules, as well as executive functions such as inhibitory control, and is used to analyze higher-order cognitive functioning in the frontal lobe of the brain. This tool is particularly valuable in assessing ADHD, revealing specific impairments in executive functioning (Chang et al., 2022).

The Trail Making Test (TMT) measures transferential cognitive flexibility and visual attention and is divided into two parts: numbers and numbers and letters. Participants are required to connect numbers or numbers and letters in sequence, and the time and quality of completing the task can reflect their cognitive flexibility and ability to perform complex tasks (Liang et al., 2022).

The Flanker Task assessed CF and attention allocation by asking participants to make choices based on the direction of the fish in the center of the school. This task measured participants’ ability to maintain goal-directed behavior in the face of distracting information and their flexibility to adjust strategies when task rules changed (Benzing et al., 2018).

The Color Span Backwards Task assessed visual WM by asking participants to memorize and repeat the sequence of different colored coins in reverse order (Benzing et al., 2018). It tested their memory capacity and attentional control by progressively increasing the number of items to be memorized.

The Tower of London Test assessed WM and planning skills by requiring participants to move a colored ball through a minimum number of steps to match a target pattern. This task required strategic planning, forward thinking, and problem-solving skills (Liang et al., 2022).

The N-Back Test assessed WM. In this task, participants were required to compare the consistency of the currently presented stimulus with the previously presented stimulus. As the number of backtracking steps increased, the difficulty of the task increased accordingly, allowing us to measure an individual’s ability to maintain and process past information (Nejati and Derakhshan, 2021).

### 3.5 Outcome measure of executive functions

IC in EF was reported in 9 RCT studies using EF as an outcome indicator. After AE intervention in trials using Stroop Task, Flanker Task, and Go-No-Go Task before and after the intervention in the control and experimental groups of eight studies showed improvement in raw score or response time for IC (Chang et al., 2012, 2022; Pan et al., 2015, 2016; Memarmoghaddam et al., 2016; Benzing et al., 2018; Benzing and Schmidt, 2019; Liang et al., 2022); however, an improvement in raw score for IC was shown in the study by Nejati and Derakhshan (2021), but no significant difference was found in RT (Nejati and Derakhshan, 2021).

For CF in EF, seven studies performed Flanker Task, Wisconsin Card Sorting Test, and Trail Making Test. After comparing the differences between the experimental and control groups before and after showed a significant improvement in raw score or response time (Chang et al., 2012; Pan et al., 2015; Benzing et al., 2018; Benzing and Schmidt, 2019; Nejati and Derakhshan, 2021; Chang et al., 2022). In the trial of Liang et al. (2022), response time improved, but raw score had no significant effect.

Four studies on WM used the Color Span Backwards Task, N-Back Test, and Tower of London Test for pre- and post-experimental testing of experimental and control groups (Benzing et al., 2018; Benzing and Schmidt, 2019; Nejati and Derakhshan, 2021; Liang et al., 2022). Improvements in both raw score and response time for WM were shown in Liang et al. (2022) and Nejati and Derakhshan (2021), but in Liang et al.’s (2022) study, raw score improvement was slight. However, in two other studies, Benzing et al. (2018) showed no improvement in raw score in a study that performed a single 15-min training session. Benzing and Schmidt (2019) subsequently showed no significant difference in raw score before or after different groups after an 18-week trial.

### 3.6 Meta-analysis of the effect of exercise intervention

**Figure 2:**
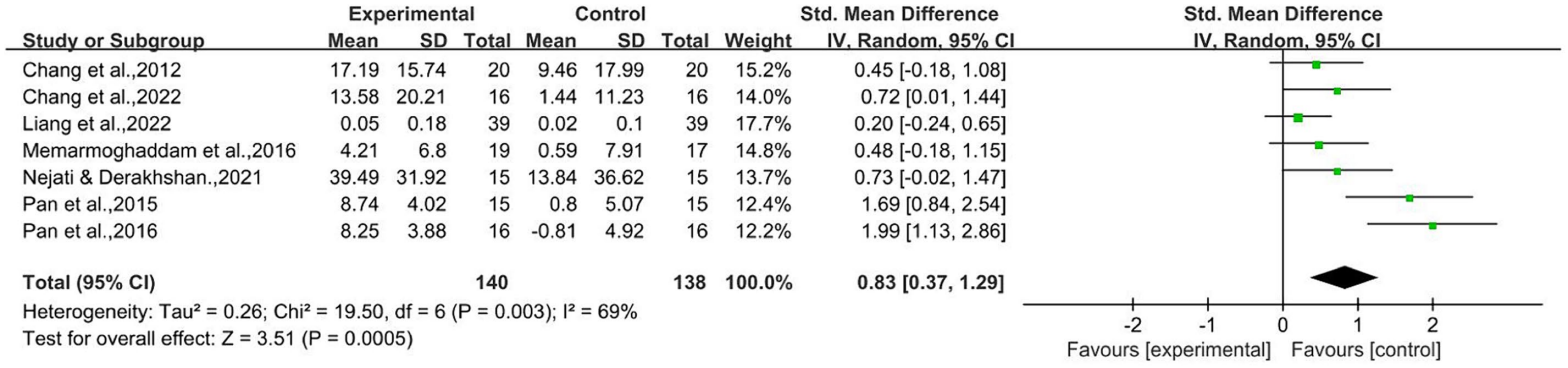
Inhibition control raw score.

A meta-analysis of seven RCT studies was performed and the outcomes were tested using the Accuracy-incongruent of Flanker Task, Color-word of Simon Task, and Go-True number raw score of Go-No-Go Task as outcome indicators (Chang et al., 2012; Pan et al., 2015; Memarmoghaddam et al., 2016; Pan et al., 2016; Nejati and Derakhshan, 2021; Chang et al., 2022; Liang et al., 2022). The effect of an AE intervention on IC in EF was analyzed in 140 children with ADHD. As shown in Figure 2, with **I*^2^* = 69% after inclusion and analyzed using a random effects model, The results of meta-analysis showed that the IC function in the EF in question showed significant improvement from before the start of the experiment to the end. Specifically, the improvement in inhibitory control functioning was significantly confirmed by statistical analysis [SMD = 0.83,95% CI (0.37–1.29), *Z* = 3.51, *p* = 0.0005]. These results suggest that the children with ADHD who participated in the study experienced significant improvements in inhibitory control.

**Figure 3:**
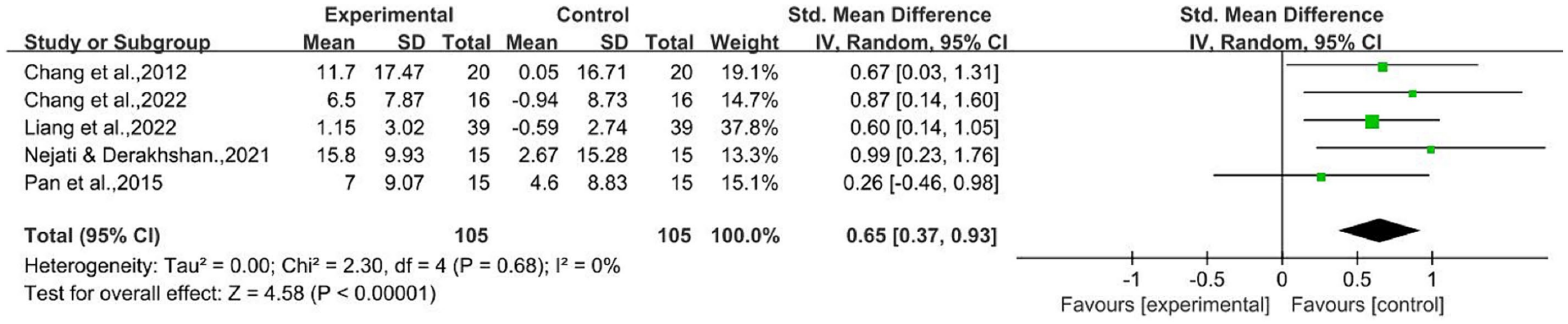
Cognitive flexibility raw score.

Five studies tested CF in EF using the Wisconsin Card Sorting Test and Trail Making Test with an experimental group involving 105 children with ADHD (Chang et al., 2012; Pan et al., 2015; Nejati and Derakhshan, 2021; Chang et al., 2022; Liang et al., 2022). Total correct or non-perseverative errors and Part A or B errors raw scores were included in the meta-analysis as outcome indicators. As shown in Figure 3, the combined statistic corresponded to **I*^2^* = 0% and thus was analyzed using a random-effects model. The results showed statistically significant differences between the two groups before and after the intervention [SMD = 0.65,95% CI (0.37–0.93), *Z* = 4.58, *p* < 0.00001], and AE had a positive improvement effect on shifting CF.

**Figure 4:**
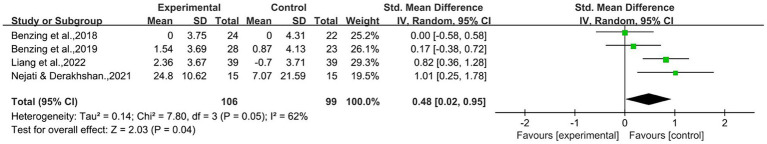
Working memory raw score.

Four studies administered AE interventions to an experimental group of 106 children with ADHD and assessed the effect of AE interventions on WM in the EF of children with ADHD using Color Span Backwards Task, N-Back Test, and Tower of London Test (Benzing et al., 2018; Benzing and Schmidt, 2019; Nejati and Derakhshan, 2021; Liang et al., 2022). Two studies demonstrated a positive effect of AE on WM (Nejati and Derakhshan, 2021; Liang et al., 2022), but two studies showed no significant difference ( *p* > 0.05) in the improvement of WM with AE (Benzing et al., 2018; Benzing and Schmidt, 2019). Updating, accuracy, and total move scores raw scores from N-Back Test and Tower of London Test used in the four studies were included in the meta-analysis, showing **I*^2^* = 62%, using a random effects model. As shown in Figure 4, the results showed statistically significant ( *p* = 0.04), indicating that AE improved WM in children with ADHD with EF [SMD = 0.48, 95% CI (0.02–0.95), *Z* = 2.03, *p* = 0.04].

### 3.7 GRADE quality evaluation

Following the GRADE criteria, we conducted an evidence-based assessment of aerobic exercise interventions on three aspects of core functioning (inhibitory control, working memory, and cognitive flexibility) in executive functioning in children with ADHD. As shown in Table 4, aerobic exercise demonstrated moderate-quality evidence support for improving inhibitory control, cognitive flexibility, and working memory in 6- to 12-year-old children with ADHD. Although “Risk of bias” was rated as “yes,” performance on the other ratings was “no,” supporting the effectiveness of the intervention.

**Table 4:** GRADE evidence summary results.

Outcomes	Presence of downgrading item of GRADE					Quality of evidence
	Risk of bias	Inconsistency	Indirectness	Imprecision	Publication bias	
Inhibitory control	Yes	No	No	No	No	Moderate
Working memory	Yes	No	No	No	No	Moderate
Cognitive flexibility	Yes	No	No	No	No	Moderate

Risk of bias: in the RCTs assessed, there were problems with the randomization process and opaque information about allocation concealment and assessor/subject blinding, resulting in potentially biased results; inconsistency: there were slight differences in point estimates among the studies, but 95% confidence intervals overlapped across all the studies and tests for heterogeneity (e.g., *I*^2^ statistic) results were not significant, indicating good agreement between results; indirectness: aerobic exercise assessed directly targeted core executive functions in children with ADHD, directly correlating with the study results, and did not show indirectness with the study results; imprecision: overall sample size was over 300, and there were no wide 95% confidence intervals for effect sizes, ensuring the statistical precision and reliability of the results; publication bias: Egger’s test showed a *p*-value greater than 0.05 and the study involved a comprehensive search of several international databases with no unpublished studies.

With regard to the “Risk of bias” rating of “Yes,” the “Risk of bias” rating of “Yes” was mainly due to the fact that the interventions involved were not evaluated. “, mainly because the studies involved had deficiencies in the randomization process and in the implementation of blinding. This included a lack of transparency in the method of concealment of randomization in some studies and the fact that knowledge of the grouping by the evaluator or participants may have affected the objectivity of the study; “No” for “Inconsistency,” although there was some variability among the individual studies. In the case of Inconsistency, although there was some variability between individual studies, the overall trend was consistent, suggesting that aerobic exercise has a positive impact on improving executive function in children with ADHD. The results of heterogeneity tests (e.g., *I^2^* statistic) support the consistency of the findings; for “Indirectness,” our analyses directly addressed the target population and intervention of the study, i.e., children aged 6–12 years with ADHD. Our analysis directly addressed the target population and intervention of the study, i.e., children aged 6–12 years with ADHD, so there was no question of indirectness; for “Imprecision,” we rated “No” because, although some of the study samples were small, the overall sample size was sufficient to ensure that the results were representative and accurate; “For “Publication bias,” we conducted an Egger’s test, which showed that the *p*-value was greater than 0.05, indicating that no significant publication bias was detected in the existing studies. This result reduces the concern about possible bias in the results of the studies and strengthens the credibility of the evidence.

### 3.8 Moderator analysis

As shown in Table 5, to analyze the potential role of other moderating variables, subgroup analyses based on Intervention type (SAE or CAE), Intervention intensity (MI, MVI), Intervention cycle (WK), Intervention time (MIN) and Use of medication were performed using Revman 5.4. to explore whether there was an interaction between IC and CF subgroups and effect sizes with subgrouped variables, respectively. Subgroup analyses were performed on the categories of variables based on Intervention type (SAE or CAE), Intervention intensity (MI, MVI), Intervention cycle (WK), Intervention time (MIN) and Use of medication.

**Table 5:** Result of subgroup analysis.

Sub-group classification	Level	IC					CF				
		*N*	SMD	95%CI	*Z*	*P*	*N*	SMD	95%CI	*Z*	*P*
Intervention type	SAE	134	1.17	[0.44,1.90]	3.15	0.002	102	0.60	[0.20,1.00]	2.96	0.003
	CAE	144	0.38	[0.05,0.71]	2.23	0.03	108	0.70	[0.31,1.09]	3.51	0.0004
Intervention intensity	MI	134	1.17	[0.44,1.90]	3.15	0.002	102	0.60	[0.20,1.00]	2.96	0.003
	MVI	114	0.29	[−0.08,0.66]	1.54	0.12	78	0.60	[0.14,1.05]	3.93	0.010
Intervention cycle	<6wk	70	0.56	[0.08,1.04]	2.31	0.02	70	0.80	[0.31,1.29]	3.20	0.001
	6-12wk	208	0.96	[0.29,1.62]	2.82	0.005	140	0.58	[0.24,0.92]	3.36	0.0008
Intervention time	15-50 min	70	0.56	[0.08,1.04]	2.31	0.02	70	0.80	[0.31,1.29]	3.20	0.001
	60-90 min	208	0.96	[0.29,1.86]	2.82	0.0005	140	0.74	[0.40,1.09]	4.23	0.0001
Use of medication	YES	134	1.17	[0.44,1.90]	3.15	0.002	–	–	–	–	–
	NO	114	0.29	[−0.08,0.66]	1.54	0.12	–	–	–	–	–

*N*, number; SAE, single aerobic exercise; CAE, combination aerobic exercise; MI, moderate intensity; MVI, moderate to vigorous intensities; WK, week; MIN, minutes.

AE treatment modality was influenced by the type of intervention. Among them, single aerobic exercise (single aerobic exercise) had the best effect on the improvement of IC in EF in children with ADHD SMD = 1.17, 95% CI (0.44–1.90), *Z* = 3.15, *p* = 0.002; the effect of single aerobic exercise and combination aerobic exercise on the improvement of shifting CF in participants did not differ significantly, the effect size of improvement in the single aerobic exercise group was SMD = 0.60,95% CI (0.20–1.00), *Z* = 2.96, *p* = 0.003; the effect size of improvement in the combination aerobic exercise group was SMD = 0.70,95% CI (0.31–1.09), *Z* = 3.51, *p* = 0.0004.

In the subgroup analysis of intervention intensity, inhibition control was influenced by moderate intensity and moderate to vigorous intensities, moderate intensity showed the best improvement SMD = 1.17, 95% CI (0.44–1.90). In addition, the effects of moderate intensity and moderate to vigorous intensities on shifting CF were the same: the effect size of moderate intensity group SMD = 0.60,95% CI (0.20–1.60), and the effect size of moderate to vigorous intensities group SMD = 0.60,95% CI (0.20–1.60), (0.20–1.00), *Z* = 2.96, *p* = 0.003; the effect size SMD = 0.60,95% CI (0.14–1.05), *Z* = 3.93, *p* = 0.010 in the moderate to vigorous intensities group.

IC was influenced by the intervention cycle, and AE interventions of 6–12 weeks performed better than those of less than six weeks SMD = 0.96,95%CI (0.29–1.60), *Z* = 2.82, *p* = 0.005. In contrast, for shifting CF improvement, AE interventions of less than six weeks performed better SMD = 0.80,95% CI (0.31–1.29), *Z* = 3.20, *p* = 0.001. The effect of aerobic exercise intervention was influenced by the intervention time, with 60–90 min being more effective in improving EF function. Specifically, the 60-90 min clock improved IC better than the 15-50 min intervention time, SMD = 0.96,95%CI (0.29–1.86), *Z* = 2.82, *p* = 0.0005. Meanwhile, shifting CF was influenced by intervention time, with an intervention time of 15-50 min SMD = 0.80,95%CI (0.31–1.29), *Z* = 3.20, *p* = 0.001 was similar to the improvement of aerobic exercise with 50-90 min SMD = 0.74,95%CI (0.40–1.09), *Z* = 4.23, *p* = 0.0001.

In a subgroup analysis of an AE intervention, significant improvement was observed in the IC of children with ADHD taking medication by AE SMD = 1.17,95%CI (0.44–1.90), *Z* = 3.15, *p* = 0.002, whereas there was no statistically significant improvement in the IC of children with ADHD not taking medication by AE SMD = 0.29, 95%CI (−0.08–0.66), *Z* = 1.54, *p* = 0.12.

### 3.9 Meta-regression analysis

In order to examine the effect of aerobic exercise intervention on executive function in children with ADHD, after combining statistics regarding core functions IC and CF in EF, we performed subgroup analysis and meta-regression analysis on intervention duration, intensity, cycle, time, and use of medication variables. As shown in the Table 6, the results showed that SAE ( *β* = 0.867, *p* < 0.001), CAE ( *β* = 0.521, *p* = 0.021), MI ( *β* = 0.928, *p* < 0.001), MVI ( *β* = 0.418, *p* = 0.021), less than 6 weeks ( *β* = 0.697, *p* < 0.001), 6–12 weeks ( *β* = 0.804, *p* < 0.001), 15–50 min ( *β* = 0.697, *p* < 0.001), 60–90 min ( *β* = 0.894, *p* < 0.001) and the use of medication ( *β* = 0.41, *p* = 0.002) were significantly associated with EF. While no medication use ( *β* = 0.881, *p* = 118) was not significantly associated with EF. In addition, meta-regression analyses were performed to determine whether the intervention duration, intensity, cycle, time, and use of medication variables had an effect on the various categories of indicators in the Table 7. The regression results showed that type of intervention ( *β* = −0.294, Q = -0.94, df = 1, *p* = 0.372), intervention intensity ( *β* = −0.485, Q = −1.38, df = 1, *p* = 0.203), intervention cycle ( *β* = 0.075, Q = 0.22, df = 1, *p* = 0.831), intervention time ( *β* = 0.159, Q = 0.50, df = 1, *p* = 0.629), and use of medication ( *β* = −0.850, Q = **−**1.55, df = 1, *p* = 0.196) did not have a significant relationship on the effect of EF.

**Table 6:** Results of subgroup analysis after EF merger.

Model	Covariate	*β*	Lower 95%CI	Upper 95%CI	*P*	R^2^ analog	Tau^2^
1	Intervention type					−0.007	0.121
	SAE	0.867	0.494	1.240	<0.001		
	CAE	0.521	0.478	1.031	0.004		
2	Intervention intensity					0.208	0.133
	MI	0.928	0.481	1.375	<0.001		
	MVI	0.418	0.131	0.705	0.004		
3	Intervention cycle					−0.260	0.151
	<6wk	0.697	0.354	1.039	<0.001		
	6-12wk	0.804	0.398	1.21.	<0.001		
4	Intervention time					−0.235	0.140
	15-50 min	0.697	0.354	1.039	<0.001		
	60-90 min	0.894	0.492	1.297	<0.001		
5	Use of medication					0.300	0.282
	YES	1.202	0.455	1.950	0.002		
	NO	0.881	−0.075	0.665	0.118		

SAE, single aerobic exercise; CAE, combination aerobic exercise; MI, moderate intensity; MVI, moderate to vigorous intensities.

**Table 7:** ES meta-regression analysis results.

ES	No. of studies/comparisons	Coef. ( *β* )	Std. err.	95% conf. interval		Test for between-groupheterogeneity		
						Q-value	df (Q)	*p*-value
Intervention type	12	−0.294	0.314	−0.994	0.406	−0.94	1	0.372
Intervention intensity	10	−0.485	0.350	−1.294	0.323	−1.38	1	0.203
Intervention cycle	12	0.075	0.335	−0.672	0.819	0.22	1	0.831
Intervention time	12	0.159	0.320	−0.553	0.872	0.50	1	0.629
Use of medication	6	−0.850	0.549	−2.374	0.673	−1.55	1	0.196

## 4 Discussion

This review examined the effects of AE on executive function in children with ADHD aged 6–12 years. After analyzing the outcome data from nine RCT trials, the findings showed statistically significant differences in IC, CF, and WM between the experimental and control groups before and after the experiment, indicating a positive effect of an AE intervention on the improvement of EF in ADHD patients. In addition, this study found that the effects of IC and CF were influenced to some extent by the variables of intervention type, intervention intensity, intervention cycle, intervention time, and use of medication.

Several previous meta-analyses have reported positive improvements from EF-focused exercise interventions in patients with ADHD (Tan et al., 2016; Liang et al., 2021); in a review by Liang et al. (2021), children and adolescents with ADHD were studied and evidence was found for improvements in IC and CF with AE intervention in a subgroup (Tan et al., 2016; Liang et al., 2021) patients aged 3–25 years, exercise was found to improve IC and WM. However, because of differences in exercise type and age characteristics, the effect of AE on the improvement of EF in children with ADHD cannot be accounted for. We included only relevant studies of children aged 6–12. In the meta-analysis, seven studies testing IC using the Flanker Task, Stroop Task, and Go-No-Go Task showed significant improvements in EF after combining the effect sizes. In the tests of CF, five studies using the Wisconsin Card Sorting Test and Trail Making Test task on shifting CF showed a positive effect on EF improvement after combining effect sizes. Further, in the four studies using the Color Span Backwards Task, N-Back Test, and Tower of London Test on WM, AE was also shown to improve WM in children with ADHD after combining effect sizes. Hence, AE resulted in an improvement in overall EF in children with ADHD.

Of the nine included studies, no adverse events were reported in the experimental group participating in AE. This finding has important implications for clinical practice, suggesting that AE may not only be beneficial for improving EF in children with ADHD, but also have a favorable safety profile, possibly making AE a valuable and safe intervention. Our effect sizes for IC, CF, and WM were statistically significant after combining the statistics. However, some studies still provided non-significant improvements or negative results. For example, Liang et al. (2022) showed no significant improvement in accuracy-incongruent before and after the experiment after testing IC using the Flanker Task in their study; Benzing et al.’s study in WM for two consecutive years with 1-week and 8-week interventions on color span showed no significant improvement. Analyses of the sum of correct responses in the regression task showed no significant difference and the study results were negative (Benzing et al., 2018; Benzing and Schmidt, 2019). Notably, all nine studies showed that AE was effective in improving IC.

In addition, in the subgroup analysis it was found that variables such as different types of exercise, intensity of intervention influenced the effect of inhibition and cognitive flexibility improvement. Also we found in the subgroup analysis after combining IC, CF that the intervention type, intensity, duration, period, and the use of medication showed to be significant in the variables, and the effect size of a single exercise program, moderate intensity, 60–90 min, 6–12 weeks, and the use of medication was greater than the effect size of the other subgroups, but no between-group differences were observed in their meta-regression analyses, which may be related to the sample size, study design heterogeneity, different implementation methods, or measurement error. Therefore, the results may be biased and this relative advantage should be interpreted with caution.

In terms of exercise type improvement, in terms of motor type improvement, overall SAE improved a greater effect size than CAE. Neudecker et al. (2019) used a systematic review of research methods to conclude that CAE had the greatest effect on ADHD children’s cognitive, behavioral, Improvements in ADHD symptom presentation and fine motor accuracy were mainly evident in male and female participants (Choi et al., 2014; Pan et al., 2014), but the study lacked data support in this regard. However, in our data-supported analysis, the opposite conclusion was reached, finding that SAE was superior to CAE in improving inhibitory control, and that SAE was more effective in improving executive functioning in children with ADHD; in addition to this, it is interesting to note that out of the nine studies included, both the experimental and the control groups in the study by Nejati and Derakhshan (2021) used an aerobic exercise intervention. The difference was that the experimental group used Exercise for cognitive improvement and rehabilitation (EXCIR) cognitive integrated category of aerobic exercise, which consisted of 12 exercises such as color jumping, arrow jumping, number jumping, pattern walking, slow walking, and limb movement, while the control group had a single event category of Aerobic exercise running, both groups showed improvement in executive function, but the combined aerobic exercise with cognitive category was better than the running program improvement. This result may be due to the fact that the EXCIR cognitive category of aerobic exercise is designed with the idea of combining physical activity with cognitive challenges, which not only promotes physical fitness but also enhances executive functioning through an exercise component with progressive cognitive demands (Nejati and Derakhshan, 2021). This leads us to subsequently place more emphasis on exploring the effects of combining physical activity and cognitive challenge in an integrated class of aerobic exercise to improve executive function when evaluating interventions for children with ADHD.

A single session of moderate to vigorous physical activity (MVPA) was found to be more effective for EF enhancement in children with ADHD in a review by Chueh et al. (2022), who also concluded that age was unlikely to be a factor influencing the results, and which included three studies of 11–16 year old adolescents among them. However, in our review only children aged 6–12 years were included and intervention intensity was found to influence the improvement in inhibitory control, as evidenced by a better improvement in overall executive functioning gains with moderate intensity than with moderate to vigorous intensity. We emphasized the importance of matching children’s developmental stage and individual differences, and that for children aged 6–12 years, moderate-intensity physical activity provides a more optimal balance of stimulating cognitive and physical development while maintaining children’s engagement and interest, ultimately leading to better improvement in inhibitory control.

Aerobic exercise was shown to improve EF effect sizes more significantly over the intervention period of 6–12 weeks, with better inhibitory control improvements than less than 6 weeks; however, improvements in switching cognitive flexibility were shown to be better at less than 6 weeks. The better improvement in inhibitory control may be due to the fact that long-term exercise promotes functional connectivity and the overall efficiency of neural networks in regions of the brain associated with executive function, especially in areas such as the anterior cingulate gyrus, occipital lobe, and frontal lobe. Long-term exercise also increases the nutrient supply to neurons in the hippocampal gyrus, which enhances memory and cognitive function (Song et al., 2023; Wang et al., 2023). For the enhancement of cognitive flexibility, especially over a period of less than 6 weeks, it may be attributed to a rise in physiological activation levels triggered by aerobic exercise. This rise is primarily characterized by an increase in key neurotransmitters such as dopamine and norepinephrine in the body, which play a positive and direct role in improving executive function (Cantelon and Giles, 2021). Additionally, this result may be due to the fact that in trials of less than 6 weeks, Chang et al.’s study was only tested after running on a treadmill for One session of 30 min, which may have had an impact on the overall amount of effect.

Finally, the effect size of 60–90 min aerobic exercise to improve EF was more significant among the different intervention durations, and the improvement of inhibitory control showed the best effect of 60–90 min, probably due to the fact that sustained aerobic exercise can increase the level of cortisol, which is important for inhibiting the over-activation of the submandibular body and contributes to the improvement of inhibitory control (Heijnen et al., 2016). Improvements in shifting cognitive flexibility showed little difference between 15–50 min and 60–90 min improvements. The good effects of 15–50 min and 60–90 min exercise on shifting cognitive flexibility improvements may be related to the pattern of activity in which the brain makes adjustments, enhancing bilateral superior frontal, middle frontal, and superior lobular activity in the performance of cognitive flexibility tasks that typically trigger activity in prefrontal and parietal regions (Jamadar et al., 2010; Boucard et al., 2012).

It was concluded that with medication in children with ADHD, the medication acts directly on the organs for metabolism and reduces the duration of treatment, whereas AE regulates the metabolic level of the body and provides a physiological basis for the efficient transmission of neural signals (Li et al., 2016), thus making AE more effective in children with ADHD taking medication. However, this result may be biased due to differences in the number of studies included in this meta-analysis, and more studies will be included subsequently to support this conclusion.

For clinicians, AE—as a non-pharmacologic adjunctive therapy—can improve EF in children with ADHD, particularly in the areas of IC and CF. This provides a new clinical intervention that can be combined with 15–50 min of moderate-intensity AE in a treatment program to supplement medication and perhaps improve treatment outcomes. For researchers, the positive effect of AE in improving EF in children with ADHD suggests that more rigorous and standardized RCTs are needed to validate these findings and further determine how the optimal duration, intensity, and frequency of intervention; intervention period; and medication use affect AE to provide more precise theoretical guidance for clinical practice.

## 5 Strengths and limitations

The strengths of this study lie in the construction of our conceptual model, the selection of the study population, and the experimental design. First, the conceptual model was methodologically oriented toward AE, for which a “conceptual model of AE intervention for children with ADHD in executive function” was proposed. The concept of AE was defined, AE studies of single and combined exercise types were included, and AE studies of moderate intensity or moderate to vigorous intensity were included. Second, the study population was selected from children aged 6–12 years with a diagnosis of ADHD, and the mean age of children with ADHD in the nine included studies was 9.31 ± 1.33 years. Finally, for the choice of experimental design, we included only RCT experimental studies to ensure the quality level of the included studies.

It is understood that relative to other studies of exercise interventions for EF in children with ADHD. This study is the first systematic evaluation and meta-analysis of the effects of AE interventions on EF in children with ADHD aged 6–12 years, but there are still several limitations in the study. First, the included studies needed to comply with the RCT study design, while the selection in age made the number of compliant studies limited, which may lead to insufficient evidence for the results of the analysis. Second, boy participants accounted for 80% of the total participants in the analyzed studies, which prevented us from developing an exploration of the effect of gender differences on the improvement of EF. In addition, the lack of girls in five studies may have also biased our findings (Pan et al., 2015, 2016; Memarmoghaddam et al., 2016; Nejati and Derakhshan, 2021). Finally, studies have focused primarily on the analysis of reactive cognitive flexibility rather than spontaneous cognitive flexibility, and this selective focus has its limitations, one of the included studies did not report medication use (Nejati and Derakhshan, 2021), and the remaining studies did not have an unmedicated control group and also lacked studies that controlled for the type and dose of medication used in children with ADHD (Pan et al., 2015). The difference in the number of studies makes it difficult to analyze the effect of improvement in CF, and WM in children with ADHD with or without medication.

## 6 Conclusion

Aerobic exercise intervention modality can effectively improve executive function in children with ADHD. In the improvement of executive function, a single aerobic exercise class is more conducive to the improvement of executive function in children with ADHD; the intervention period is 6–12 weeks, the intervention time is 50-90 min, moderate intensity aerobic exercise intervention is more conducive to the improvement of executive function, and the improvement effect is obvious in children with ADHD who are taking medication. It was particularly valuable in improving inhibitory control and cognitive flexibility. However, considering the differences in the included studies, methodological quality, intervention period, and medication use, additional, rigorous, and standardized RCTs are needed to define specific intervention protocols further.

## Data availability statement

The original contributions presented in the study are included in the article/supplementary material, further inquiries can be directed to the corresponding authors.

## Author contributions

GY: Writing – review & editing, Writing – original draft, Software, Methodology, Formal analysis, Data curation. QL: Writing – review & editing, Validation, Supervision, Resources, Methodology, Formal analysis, Data curation, Conceptualization. WW: Writing – review & editing. WL: Writing – review & editing. JL: Writing – review & editing, Validation, Supervision, Resources, Project administration, Formal analysis, Conceptualization.
